# Monte Carlo simulation studies on scintillation detectors and image reconstruction of brain-phantom tumors in TOFPET

**DOI:** 10.4103/0971-6203.56083

**Published:** 2009

**Authors:** Nagendra Nath Mondal

**Affiliations:** High Energy Physics Group, 1/AF Bidhannagar, Kolkata 700064, India. E-mail: nagendra.mondal@veccal.ernet.in

**Keywords:** GEANT, Monte Carlo, phantom, PV method, scintillators, TOFPET

## Abstract

This study presents Monte Carlo Simulation (MCS) results of detection efficiencies, spatial resolutions and resolving powers of a time-of-flight (TOF) PET detector systems. Cerium activated Lutetium Oxyorthosilicate (Lu_2_SiO_5_: Ce in short LSO), Barium Fluoride (BaF_2_) and BriLanCe 380 (Cerium doped Lanthanum tri-Bromide, in short LaBr_3_) scintillation crystals are studied in view of their good time and energy resolutions and shorter decay times. The results of MCS based on GEANT show that spatial resolution, detection efficiency and resolving power of LSO are better than those of BaF_2_ and LaBr_3_, although it possesses inferior time and energy resolutions. Instead of the conventional position reconstruction method, newly established image reconstruction (talked about in the previous work) method is applied to produce high-tech images. Validation is a momentous step to ensure that this imaging method fulfills all purposes of motivation discussed by reconstructing images of two tumors in a brain phantom.

## Introduction

The Department of Atomic Energy (DAE) of India is going to establish its first Medical Cyclotron Facility (MCF) in Kolkata with other existing facilities. It was scheduled to be commissioned by the end of 2008. MCF will primarily be used for the production of radioisotopes for symptom imaging in Single Photon Emission Tomography (SPECT) and in Positron Emission Tomography (PET), besides applications in other front line research.[1] PET is a powerful metabolic imaging technique potentially used in small size brain tumor identification. The best radiopharmaceutical [^18^F]-fluorodeoxyglucose (FDG) is widely used in clinical oncology as a positron (e^+^, an antiparticle of e^−^) emitter which concedes of high quality images. Our objective is to design a sophisticated time-of-flight positron emission tomography (TOFPET) gamma camera to utilize one of the on-time shared beam lines for innovative research. In TOFPET, “position vector (PV)”, an image reconstruction method is applied where both energy and time of collinear γ-rays are taken into account. Those γ-rays are produced due to e^+^e^−^ annihilation. The working principle of PV method is described in section 3 and can also be found elsewhere[2] and references there in.

Monte Carlo Simulation (MCS) plays a vital role in designing the TOFPET system and modeling the image of a diagnostic object as many other branches of science. The MCS of TOFPET is executed with the aid of GEANT3.21.[3] The main interests of MCS are: (i) crucial role played by GEANT3.21 in TOFPET, (ii) reconstruction of image of the diagnostic object using the newly established PV method instead of conventional image processing techniques,[2] (iii) detection efficiencies and blurring effect in the presence of a brain phantom and (iv) validation of the PV method.

Phantom is a material that has physical properties similar to biological tissues and the human body. Biological tissue equivalent phantom was developed by Ito *et al*, in Chiba University, Japan which can be classified into two major categories: the high water content tissue such as muscle, brain and internal organs and low water content tissue such as fat and bone. Energy and time resolutions of Cerium-activated Lutetium Oxyorthosilicate (LSO), Cerium doped Yttrium Aluminum Perovskite (YAP), BriLanCe 380 (Cerium doped Lanthanum tri-Bromide, LaBr_3_)[4] and Barium Fluoride (BaF_2_)[5] have already been studied for different applications considering their good time and energy resolutions, γ-ray stopping powers, shorter decay times etc. However, systematic studies and comparison among LSO, LaBr_3_ and BaF_2_ are not yet accomplished for the purposes of TOFPET.

In this study a real TOFPET system with 48 LSO scintillation detector (each size is (3φ**×3 cm^3^) and ring diameter of 80 cm is exhibited as proposed by earlier studies. Two close contact tumors are imaged by the PV method utilizing a tissue equivalent brain phantom conveying its validation.

## TOFPET Resolution

A few definitions are given below. These are particularly creditable for subsequent discussions in characterizing TOFPET.

### Energy Resolution

The energy resolution (*E*_R_) of a radiation detector emphasizes the precision with which the system can measure the energy of incident photons. In scintillation detectors the *E*_R_ is a function of the relative light output of the scintillator and other associate electronics. *E*_R_ is directly proportional to Δ*E*. To attain good image contrast it is essential to have better *E*_R_ and this can be achieved by reducing the background and scattered events. Also fine tuning of detector, data acquisition system and data analysis techniques are required.

### Time Resolution

The time resolution (*T*_R_) of a TOFPET is one of the crucial parameters for image reconstruction with least blurring effects (an effect dominated by random and scattered events). The full width at half maximum (fwhm) of a TOF distribution is often used as a measure of the overall timing uncertainty and is called the time resolution. However, the use of very fast scintillators such as CsF allows one to have a *T*_R_ ~ 400 ps[6] and or BaF_2_ ~ 120 ps.[5]

### Spatial Resolution and Resolving power

Spatial resolution (*S*_R_) or more accurately the resolving power (*R_P_*) is the other important parameter in TOFPET system. *R_P_* is the ability of the components of TOFPET to measure the minimum resolvable distance between distinguishable objects in an image. It determines the quality of the image, and depends on the image reconstruction, data analysis and data acquisition processes. The measurement technique of *S*_R_ can be found elsewhere.[2] *R*_P_ can be expressed in the following form:

(1) $$\text{R}p=\left(\frac{\Delta \sigma }{\sigma_{or}}\right)\times 100\%$$

Where, *σ* is the standard deviation of Gaussian distribution, Δ*σ* = Average *σ* of reconstructed positions - average *σ* of original positions, and *σ*_or_ = average *σ* of the original positions. *R*_P_ attributes the blurring effect of a reconstructed image and its brief discussions are available in the results section.

## Design of TOFPET

The detection of two collinear photons depends upon the properties and geometry of the detectors. The type of detector chosen (scintillator as well as photomultiplier tube (PMT)) is a major part of the design of a TOFPET camera.

### Choice of Scintillation Crystals

A thorough investigation over commercially available scintillators is necessary for achieving better *T_R_*, *R_P_* and detection efficiency. Recently a renewed interest in TOFPET was generated based on the fast decay time and better *T*_R_ and *S*_R_ of new type of scintillators, e.g., Lu_2_SiO_5_:Ce[7] and LaBr_3_:Ce, which offer high sensitivity, low dead time and a good time and energy resolutions. These lead to the reduction of random count rate (*R_d_*_1_*_d_*_2_ = 2*τN_d_*_1_*N_d_*_2_, between two detectors) and scatter contribution.[8]

### Monte Carlo simulation of TOFPET

#### Array of detectors

**Figure 1:**
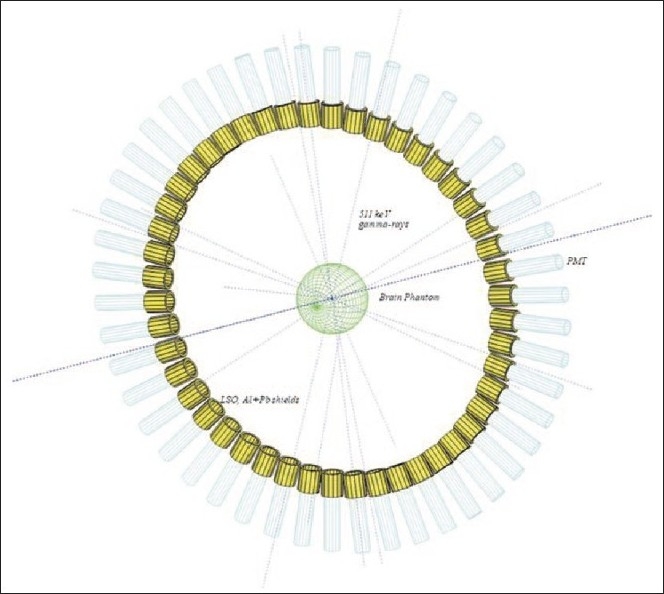
A TOFPET spectrum is illustrated. It is obtained by MCS based on GEANT3.21. A brain-phantom is located at the center of the camera

An extensive MCS study based on GEANT3.21 has been carried out to evaluate the design of a TOFPET. From among the commercially available scintillators, LSO of size 3*ø*×3 cm^3^ is optimized. A typical spectrum of TOFPET system is shown in Figure 1. There are 48 detectors in a single ring and face-to-face distance of each pair is 80 cm (diameter of the ring). A thin Al (0.1 cm) and a thick Pb (2 cm) shields are used outside the scintillator in order to reflect scintillation light and to stop Compton scattered γ-rays and natural backgrounds. An Al (3* φ*×0.1 cm^3^) disk is also used in front of the scintillator to prevent radiation damage. Detectors are placed on the *x–y* plane perpendicular to the *z*-axis which is the direction of a patient movement. *E*_R_ and *T*_R_ are taken into account in the MCS.

The MCS can provide us not only the unique information of Compton scattered and full energy deposit of incident 511 keV γ-rays in the scintillator, but also the timing information. Therefore, image reconstruction of e^+^e^−^ annihilation points by selecting those true events and utilizing the Energy-Time (E-T) correlation[9] are very convenient. A high resolution brain PET scanner (G-PET) was developed by Karp *et al*.[10] In Table 1 comparisons among the various models of PET are shown.

**Table 1:** Comparison among the various models

*Properties*	*G-PET*	*PET Simens/CTI*	*Mondal TOFPET*
Ring diameter [cm]	42	82	80
Number of ring(s)	8	24	1
Number of detectors	36	784	48
per ring			
Crystal dimension [cm^3^]	0.4×0.4×1	0.29×0.59×3	3φ×3
Type of Crystal	GSO	BGO	LSO
Spatial resolution [cm]	0.4	0.4	<1
Time resolution [ps]	—	—	886

### Brain Phantom and Positron Source

**Figure 2:**
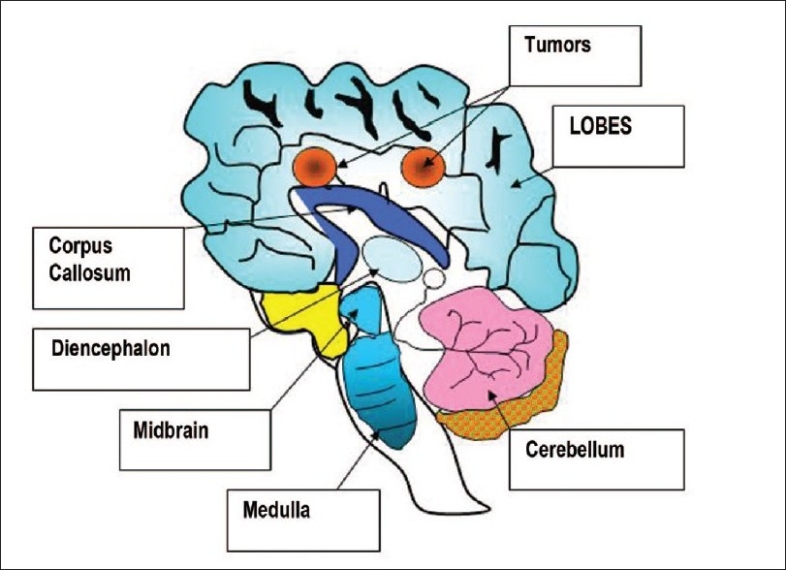
Drawing of a human brain with positions of two tumors

Let us assume that a patient has been suffering from two brain tumors closely located and those are depicted in Figure 2. The aim of this study is to model a simplest physical tissue equivalent brain phantom and validate the PV image reconstruction method. The model should be anatomically accurate and realistic to have greater impact than simply-shaped phantoms. The model should be completely 3D to be essentially realistic.

**Figure 3:**
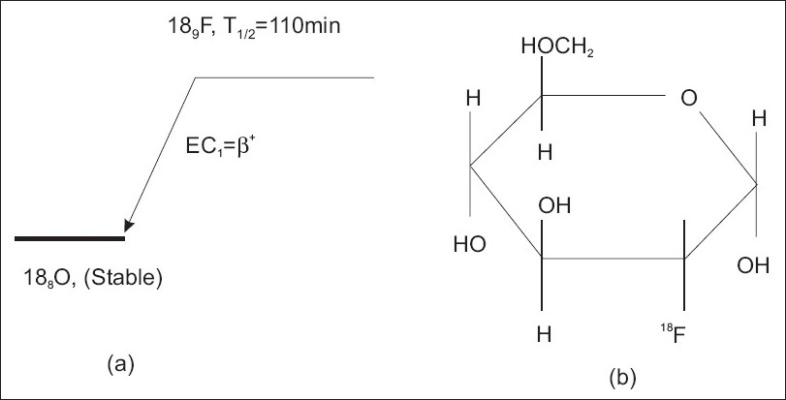
Decay scheme of ^18^_9_F (a), and molecular formula of FDG (C_6_H_11_FO_5_) (b).[11]

The assumptions taken into account in the MCS based on GEANT3.21 are as follows:

- One e^+^e^−^ annihilation event produces two collinear 511 keV photons. Those events are generated uniformly in 4π Gaussian distribution.
- Principal constituents of human head are: Skull, Brain and Water.
- Molecular Formula (MF) of human skull is C_20_H_22_N_8_O_5_. Assuming a 70 kg adult patient, volume of spherical skull (3D) is determined to be 234.99 cm^3^ and density 10.3 g/cm^3^.
- DNA (molecular weight (Mwt): 475.299 g/mol and MF: C_15_H_26_O_13_P_2_) and RNA (Mwt: 603.277 g/mol and MF: C_15_H_26_O_19_P_3_) are the main constituents of human brain and density of an adult brain is calculated to be 0.621 g/cm^3^ (person to person it may vary). RNA and DNA are uniformly distributed in mass proportion inside the spherical skull.
- A typical dose of FDG used in an oncological scan is 200 - 400 MBq for an adult human and very useful for brain tumor identification. Biochemical processes can be traced precisely and a patient can be diagnosed several times in a day due to its shorter half-life. The decay scheme of ^18^_9_F and structural formula of FDG are given in Figure 3.
- The molecular mass of C_6_H_11_FO_5_ is 182.15 g/mol and its density in the tumor site is 7.00 g/cm^3^ and Poissionian distribution of e^+^.

### PV Image Reconstruction Method in TOFPET

Instead of the conventional most likely position method[12] the PV method is applied for the image reconstruction. It is a new method of position reconstruction in TOFPET described elsewhere.[2] Position conversion factors (useful for reinstatement of e^+^e^−^ annihilation points) of this TOFPET system are determined (these values differ from system to system). General notions of image reconstruction equations in two dimensions (2D) are:[2]

(2) $$X=\sum_{i=1}^{n/2}\left[\left\{T_{i}\left|\cos\left(i-1\right)\theta \right|-T_{i+n/2}|\cos\left(i+n/2-1\right)\theta |\right\}\times C/2-c\right]/\left(2m\right)$$

and

(3) $$Y=\sum_{i=1}^{n/2}\left[\left\{T_{i}\left|\sin\left(i-1\right)\theta \right|-T_{i+n/2}|\sin\left(i+n/2-1\right)\theta |\right\}\times C/2-c\right]/\left(2m\right)$$

Where, *T_i_* and *T_i+n/2_*: TOF of collinear photons, θ=2π/*n*: angle between the neighboring detectors, *n*: even number of detectors (here n=48, see figure 1), C: speed of light, c and m respectively are intercept and slope of the fitted line of the spectrum where reconstructed versus real positions are plotted.

## Results and Discussion

### Spatial Resolution and Detection Efficiency

To procure realistic MCS, the simulated energy distribution of 511 keV photons was considered Gaussian. Time and energy resolutions of different scintillators incorporated in this study are shown in Table 2. Equal number of annihilation events (1×10^6^) are generated in each case. *S*_R_s with statistical errors (estimated from the fitting parameters) along the x-axis and detection efficiencies (*D*_ε_) of each scintillator are estimated without TOF window and brain phantom settings. Those results are shown respectively in columns 4 and 5 in table 2 and outcomes are obtained considering the point source distribution of e^+^e^−^ and from the detected 511 keV events in coincidence.

Similarly the effect of TOF window setting (1 ns) on D_ε_s and S_R_s of different scintillators can be noted easily from the Table 3 where similar T_R_ and E_R_ of table 2 are applied and coincidence events are selected using the energy-time (E-T) correlation[9] (cut of energy conservation: 950≤(*E*_1_ + *E*_2_)≤1050 keV and lower energy cut of γ-rays is 10 keV).

**Table 2:** Comparisons of Scintillators without Setting Time Window

*Name of Scintillators*	*T_R_ (ps)*	*E_R_ (at 511 keV)*	*S_R_ ± error, along x-axis (cm)*	*Dε (%)*
LSO(3φ×3 cm^3^)	886 [4]	9.1%	3.63±0.08	0.65
LaBr_3_(3φ×3 cm^3^)	576 [4]	3.9%	4.59±0.33	0.15
BaF_2_(3φ×3 cm^3^)	120 [5]	4.3%	3.07±0.10	0.20

**Table 3:** Comparisons of Scintillators with Setting of Time Window

*Name of Scintillators*	*Generated events*	*Coincidence 511 keV*	*S_R_ ± error, along x-axis (cm)*	*Dε (%)*
LSO(3φ×3 cm^3^)	1×10^6^	2012	1.32±0.02	0.201
LaBr_3_(3φ×3 cm^3^)	1×10^6^	405	1.45±0.06	0.041
BaF_2_(3φ×3 cm^3^)	1×10^6^	647	1.35±0.04	0.065

It can be perceived from Tables 2 and 3 that D_ε_s and S_R_s respectively are decreased and improved about three times. Although T_R_ and E_R_ of LSO are inferior and the S_R_s are changed significantly among the scintillators of the same order but D_ε_s of LSO increased about five and three times than those of LaBr_3_ and BaF_2_ respectively. The orders remain almost the same when time window is considered. There seems to be better execution in LSO due to its excellent photon yield (32 photons /keV) and best stopping power of γ-rays (because of the highest atomic density, 7.4 g/cm^3^). The number of detectors may be increased by adding more rings on both sides of the present TOFPET system [Figure 1] for the improvement of D_ε_. It is also observed that coincidence D_ε_s increases 8–10 times by increasing the crystal size from 1*φ*×3 cm^3^ to 3*φ*×3 cm^3^. At the same time, S_R_s degrades about three times. Results of MCS infer that E-T correlation technique is an essential tool for the improvement of S_R_ as well as R_P_. The timing uncertainty of a TOFPET detector preludes blurring effects in the image reconstruction process of X and Y.

### Determination of R_P_ in TOFPET

**Figure 4:**
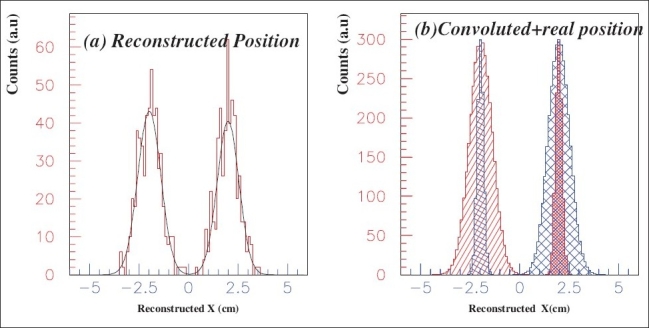
(a) Reconstructed position distribution, (b) Convoluted and real (smaller sigma) position distributions

To determine *R*_P_ and observe image resolution of this system in one dimension, let us assume two identical sources of e^+^ which are sitting 4 cm (2, 0, 0 and -2,0,0) apart from each other. Annihilation positions along the X-axis are reconstructed by using equation (2). A typical image is depicted in Figure 4.

The data is fitted with two Gaussians and convoluted using the fitting parameters after normalizing with the real spectra. Face-to-face long distance among the detectors and a few time windows make it possible to run down the blurring effects. *R*_P_ of different scintillators of this TOFPET system are determined by equation (1) and results are arranged in column 5 of Table 4. *R*_P_ of LSO attributes less blurring effects than those of other two.

**Table 4:** Resolving Power of Different Scintillators in TOFPET

*Name of Scintillators*	*σ_re,av_ of tumor, x-axis (cm)*	*σ_or,av_ of tumor, x-axis (cm)*	*Difference of σ (cm)*	*R_p_ (%)*
LSO(3φ×3 cm^3^)	0.567075	0.163874	0.403201	2.46
LaBr_3_(3φ×3 cm^3^)	0.798145	0.164270	0.633875	3.86
BaF_2_(3φ×3 cm^3^)	0.654930	0.164270	0.490660	3.00

### Validation of PV Method

**Figure 5:**
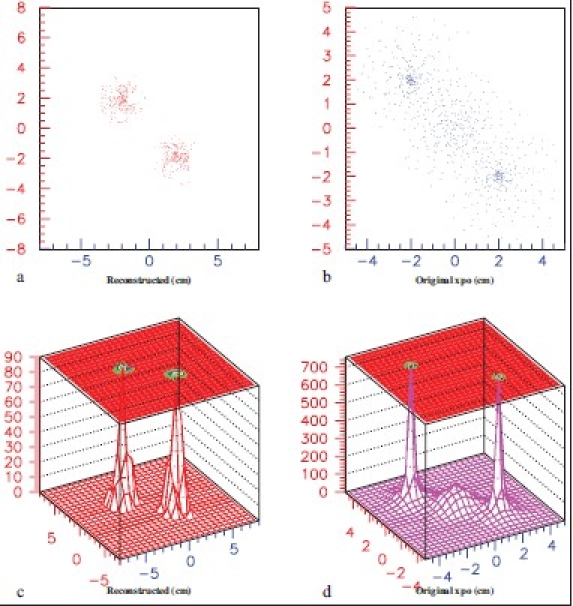
Reconstructed 2D images of two tumors (a,c), and their original 2D distributions (b,d) with backgrounds. 50% random events of 511 keV are generated at (-2,2,0), (0,0,0) and (2,-2,0) as backgrounds

One of the usages of validation is that it is a process of checking if something satisfies a certain criterion. In order to understand the validation of PV method [Figure 1] in 2D, two identical tumors at (2,-2,0 and -2,2,0) are considered in a brain phantom (stated before) and MCS is performed with backgrounds. Using equations (2) and (3) 2D images of those tumors are reconstructed and those are depicted in [Figure 5]. Reconstructed scattered (a) and its surface (c) plots show how cleanly backgrounds are suppressed by the PV method. Their original 2D scattered and corresponding surface plots [Figure 2] are shown respectively in spectra (b) and (d).

Detection efficiencies of TOFPET system in presence of a brain phantom with FDG and in absence of them are 0.11% and 0.65% respectively in the case of LSO. Blurring effect increases slightly. Results attribute that γ-rays are absorbed, scattered in different directions and attenuated in the presence of brain phantom. Perhaps the differences in results will increase when positron implantation depth, ortho-positronium and para-positronium formation effects are taken into account (none of this effects are considered here), but quality of the image might be improved. It would be very interesting if phantom model could be compared with a real data. In this method, position conversion factors are important ingredients for positioning the reconstructed position very close to its original. PV is an on-line image reconstruction method and requires a few minutes and MBq source for data acquisition, and image processing with a few Mb memories.

## Conclusion

A 2D image reconstruction of tumors in a brain phantom, performed by MCS, validates the PV method. Detection efficiencies, spatial resolutions and resolving powers of LSO, BaF_2_ and LaBr_3_ scintillators are verified. In every aspect LSO shows better performance than the other two. Proper setting of time window improves spatial resolution and resolves powers significantly. It is important to achieve better energy and time resolution of scintillation detectors to confine real number of coincidence events which has less blurring effects on the image. It may be concluded that the excellent performance of PV method shows its beauty over any conventional iterative image reconstruction technique by saving huge computational time and memory.
